# Effects of interventions on trajectories of health-related quality of life among older patients with hip fracture: a prospective randomized controlled trial

**DOI:** 10.1186/s12891-016-0958-2

**Published:** 2016-03-03

**Authors:** Ming-Yueh Tseng, Jersey Liang, Yea-Ing L Shyu, Chi-Chuan Wu, Huey-Shinn Cheng, Ching-Yen Chen, Shu-Fang Yang

**Affiliations:** Department of Nursing, Meiho University, 23 Pingguang Road, Neipu, Pingtung, 91202 Taiwan; School of Public Health, University of Michigan, 1415 Washington Heights, M3007 SPH II, Ann Arbor, MI 48109 USA; Institute of Gerontology, University of Michigan, 1415 Washington Heights, M3007 SPH II, Ann Arbor, MI 48109 USA; School of Nursing, College of Medicine, Chang Gung University, 259 Wenhua 1st Road, Guishan District, Taoyuan, 33302 Taiwan; Healthy Aging Research Center, Chang Gung University, 259 Wenhua 1st Road, Guishan District, Taoyuan, 33302 Taiwan; Traumatological Division, Department of Orthopedics, Chang Gung Memorial Hospital, 5 Fu-Hsin Street, Guishan District, Taoyuan, 33305 Taiwan; Department of Internal Medicine, Chang Gung Memorial Hospital, 5 Fu-Hsin Street, Guishan District, Taoyuan, 33305 Taiwan; Section of General Psychiatry, Department of Psychiatry, Chang Gung Memorial Hospital, 5 Fu-Hsin Street, Guishan District, Taoyuan, 33305 Taiwan

**Keywords:** Health-related quality of life trajectory, Hip fracture, Comprehensive intervention, Interdisciplinary intervention, Randomized controlled trial

## Abstract

**Background:**

Health-related quality of life (HRQoL) has been used to assess subjects’ prognosis and recovery following hip fracture. However, evidence is mixed regarding the effectiveness of interventions to improve HRQoL of elders with hip fracture. The purposes of this study were to identify distinct HRQoL trajectories and to evaluate the effects of two care models on these trajectories over 12 months following hip-fracture surgery.

**Methods:**

For this secondary analysis, data came from a randomized controlled trial of subjects with hip fracture receiving three treatment care models: interdisciplinary care (*n* = 97), comprehensive care (*n* = 91), and usual care (*n* = 93). Interdisciplinary care consisted of geriatric consultation, discharge planning, and 4 months of in-home rehabilitation. Comprehensive care consisted of interdisciplinary care plus management of malnutrition and depressive symptoms, fall prevention, and 12 months of in-home rehabilitation. Usual care included only in-hospital rehabilitation and occasional discharge planning, without geriatric consultation and in-home rehabilitation. Mental and physical HRQoL were measured at 1, 3, 6, and 12 months after discharge by the physical component summary scale (PCS) and mental component summary scale (MCS), respectively, of the Medical Outcomes Study Short Form 36, Taiwan version. Latent class growth modeling was used to identify PCS and MCS trajectories and to evaluate how they were affected by the interdisciplinary and comprehensive care models.

**Results:**

We identified three quadratic PCS trajectories: poor PCS (*n* = 103, 36.6 %), moderate PCS (*n* = 96, 34.2 %), and good PCS (*n* = 82, 29.2 %). In contrast, we found three linear MCS trajectories: poor MCS (*n* = 39, 13.9 %), moderate MCS (*n* = 84, 29.9 %), and good MCS (*n* = 158, 56.2 %). Subjects in the comprehensive care and interdisciplinary care groups were more likely to experience a good PCS trajectory (*b* = 0.99, odds ratio [OR] = 2.69, confidence interval [CI] = 7.24–1.00, *p* = 0.049, and *b* = 1.32, OR = 3.75, CI = 10.53–1.33, *p* = 0.012, respectively) than those who received usual care. However, neither care model improved MCS.

**Conclusions:**

The interdisciplinary and comprehensive care models improved recovery from hip fracture by increasing subjects’ odds for following a trajectory of good physical functioning after hospitalization.

**Trial registration:**

ClinicalTrials.gov (NCT01350557)

## Background

Health-related quality of life (HRQoL) has been recommended for assessing the prognosis and recovery of patients following a hip fracture as a supplement to objective clinical indicators [1]. However, evidence is mixed regarding the effectiveness of interventions to improve the HRQoL of elders with hip fracture [2–4]; some studies found beneficial effects [2, 4] and some did not [3].

This inconsistency in intervention benefits on HRQoL might have been due to the course of changes in HRQoL after hip fracture being averaged over patients. However, temporal changes in HRQoL for community-dwelling older persons and hospitalized older patients have been shown to be heterogeneous [5–7]. For example, older community-dwelling women were found to have four distinct trajectories of HRQoL, high (19 %), high decline (22 %), intermediate (42 %) and low decline (16 %), over a period of 7 years [5]. In another study, hospitalized, frail older persons waiting for entry to residential care had extremely poor HRQoL (worse than death-equivalent) and poor (death equivalent) at both baseline and 4-month follow-up, but with some improvement over the follow-up period [6]. Also, patients who had undergone coronary artery bypass surgery were found to have “improver” and “non-improver” HRQoL trajectories for both the physical component summary scale (PCS) and the mental component summary scale (MCS) of the Medical Outcomes Study (MOS) Short Form 36 (SF-36) during the first year following surgery [7]. Because patients may have several distinct HRQoL trajectories following hip fracture, intervention effects could vary across these trajectories. In an earlier study of elderly patients with hip fracture, we developed an interdisciplinary care model consisting of geriatric consultation, discharge planning, and 4 months of in-home rehabilitation in addition to usual care and found this model effective in improving HRQoL of older persons with hip fracture [8]. Later in a different trial, we refined the interdisciplinary care model by adding management of malnutrition and depressive symptoms as well as fall prevention and 12 months of in-home rehabilitation, thus developing a comprehensive care model [9]. Comparison of the intervention effects of the comprehensive and interdisciplinary care models to usual care showed that both models improved HRQoL of older persons with hip fracture, especially physical health-related outcomes (effect size = 0.3, 95 % CI =0.02–0.58 at 12 months after discharge) [9].

Analysis of that data was limited by focusing on the average course of changes in HRQoL without exploring whether HRQoL may change along distinct trajectories and whether the intervention effects varied for distinct trajectories. Hence, two research questions remain unanswered: Do patients with hip fracture experience multiple distinct trajectories of HRQoL? If so, what are their levels and rates of change over time? What are the effects of the interdisciplinary care and comprehensive care models compared to usual care on these distinct HRQoL trajectories? To address these questions, we conducted this secondary analysis to identify the distinct courses of change in HRQoL over 1 year after hip-fracture surgery and to analyze the intervention effects on HRQoL trajectories after comprehensive and interdisciplinary care compared to usual care. In particular, we evaluated the following two hypotheses.

- Hypothesis 1 (H_1_): HRQoL, including physical and mental health-related health outcomes, during the first year after hospital discharge following hip fracture has multiple distinct trajectories that can be characterized as poor, moderate, and good [5].
- Hypothesis 2 (H_2_): Receiving the comprehensive and interdisciplinary care models increases patients’ odds of being in a good physical and good mental health trajectories over time compared with the usual care model.

## Methods

### Design and setting

Data for this research came from a randomized control trial conducted from September 2005 to July 2010 at a medical center in northern Taiwan [9, 10].

### Subjects

Patients were included in the original study by these criteria: (a) at least 60 years old, (b) hospitalized for an accidental first time, single-side simple hip fracture and receiving hip arthroplasty or internal fixation, (c) with a pre-fracture Chinese Barthel Index (CBI) score >70 at admission and able to perform full range of motion against gravity and against some or full resistance with the unaffected limb, and (d) living in northern Taiwan. Exclusion criteria were (a) severely cognitively impaired and completely unable to follow orders (determined by a score <10 [11] on the Chinese Mini-Mental State Examination [12]), or (b) terminally ill.

Of 1246 patients with hip fracture screened, 470 met the study criteria. Among the 776 patients who did not meet our criteria, 409 (52.7 %) had poor pre-fracture physical functioning, 158 (20.4 %) did not live in northern Taiwan, 95 (12.3 %) had severe cognitive impairment, 85 (10.9 %) were unable to communicate, and 29 (3.7 %) lived in a nursing home. Among the 470 patients who met our criteria, 299 agreed to participate. Of these, only 281 had at least one HRQoL assessment. Subjects who had at least one HRQoL assessment and those without any HRQoL data did not differ significantly in gender, age, type of fracture and surgery, and pre-fracture performance of activities of daily living (ADLs). All subjects were assessed for pre-fracture ADL performance before surgery and for HRQoL outcome variables at 1, 3, 6, and 12 months after discharge. The present analysis included 281 subjects (91 comprehensive, 97 interdisciplinary, and 93 usual care) for whom HRQoL data were available during the first year following discharge. The three treatment protocols are briefly described below. Details have been published [9, 10].

Based on our prior study on intervention effects of the interdisciplinary care model, a sample size of at least 90 was found sufficient to measure changes in physical function-related indicators to achieve a power of 0.80, with a significance level of 0.05, from pre-discharge to 3 months after discharge [8]. Based on a case loss of 10 % [13], we recruited 100 in each group.

### Usual care

After receiving internal fixation or arthroplasty, subjects were cared for on trauma wards. During postoperative hospitalization (around 7 days), no geriatric consultation was provided, although internal medicine consultations were occasionally made according to the subject’s condition. Physical therapy usually started on the second or third day following surgery without any home rehabilitation. Clinical follow-ups were recommended at 1, 3, 6 and 12 months following discharge.

### Interdisciplinary care model

The interdisciplinary care model included three key components: Geriatric consultation, rehabilitation, and discharge planning.

#### Geriatric consultation

Geriatric assessment was first delivered by a geriatric nurse to assess and detect potential problems. After the nursing assessment, high-risk patients, including those >80 years old, at high operative risk, with poor nutritional status, cognitive impairment or disorientation, or with unstable co-morbid conditions were further evaluated by a geriatrician who then made recommendations to the primary surgeon.

#### Rehabilitation program

Rehabilitation included in-hospital rehabilitation starting on the first day following surgery and 4 months of in-home rehabilitation, both delivered by a geriatric nurse. Based on their recovery progress, subjects receiving interdisciplinary care were advised on a six-stage progressive muscle-strength training program, which started from ankle pumping exercise, knee extension, gently bouncing jump with knee semiflexed and both feet on the floor, and gently bouncing jump with knee semiflexed and single foot on the floor. During rehabilitation, the geriatric nurse emphasized pain relief; enhancing range of motion, muscle strength and endurance; proprioception; balance challenges, as well as improving aerobic and anaerobic capacity.

#### Discharge planning

The geriatric nurse provided a structured discharge assessment of caregiver competence, resources, family function, elderly subject’s self-care ability, elderly subjects’ and their family caregivers’ need for community or long-term care services, assessment of the home environment, and referrals to community resources referrals. If needed, the nurse also suggested environmental modifications for identified barriers at home. Reminder phone calls were also made for follow-ups.

### Comprehensive care model

The comprehensive model integrated all components of the interdisciplinary care model with an enhanced rehabilitative program, fall prevention, nutrition consultation, and management of depression.

#### Rehabilitation program

Subjects in the comprehensive group received an expanded, 1-year in-home rehabilitation program. Hence, these subjects could recover sufficiently to perform exercises related to balance challenges and aerobic capacity under instruction, whereas subjects receiving interdisciplinary care were not recovered enough to perform these activities during the 4 months in-home rehabilitation.

#### Fall prevention

The geriatric nurse assessed fall risks and provided corresponding interventions at each home visit. Assessed fall-risk factors included postural hypotension, multi-medications, impaired transfer ability, poor gait or weak leg/arm muscles, environmental hazards, and knowledge deficits.

#### Nutritional consultation/education

Subjects’ nutritional status was assessed at discharge using the Mini Nutritional Assessment (MNA) scale [14, 15]. Those who scored < 17 were categorized as malnourished and were referred to a dietitian. Those who scored ≥ 17 but ≤ 23.5 were categorized as at risk of malnutrition and were referred to a geriatrician. These high-risk patients were followed up by a geriatric nurse who provided consultation according to suggestions of the geriatrician and dietician. The nurse also assessed nutrition outcomes using the MNA at each home visit.

#### Depression screening and management

Geriatric nurses assessed subjects’ depressive symptoms using the Geriatric Depression Scale short form [16, 17] before hospital discharge and at each home visit. Subjects identified as at risk were referred to a psychiatrist or psychiatric clinic for further assessment and management under agreement of the subjects. At the same time, the geriatric nurse provided individualized consultation and emotional support for these at-risk subjects.

### Measurements

#### HRQoL

Mental and physical HRQoL were measured by the PCS and MCS, respectively of the MOS SF-36, Taiwan version [18]. The SF-36 has 36 items representing eight generic health concepts: physical functioning (PF), role disability due to physical health problems (RP); bodily pain (BP); vitality (energy/fatigue) (VT); general health perceptions (GH); social functioning (SF); role disability due to emotional problems (RE); and general mental health (MH). PCS and mental MCS using norm-based (50, 10) scoring methods were calculated based on the norm of a previous study [19].

#### Pre-fracture performance of ADLs

Pre-fracture ADL performance was retrospectively assessed using the Chinese Barthel Index (CBI) before randomization and before hip-fracture surgery. The CBI, with scores ranging from 0 to 100, measures dependencies in eating, transferring, grooming, toileting, bathing, walking, climbing stairs, dressing, as well as bowel and bladder control [20]. The CBI has been shown to have satisfactory reliability and validity for assessing Taiwanese elders with hip fracture [20, 21].

### Ethical considerations

The study was approved for human subject research by the study hospital (Chang Gung Medical Foundation, Institutional Review Board; approval number: 94-422C) and was in compliance with the Helsinki Declaration and local legislation. Informed consent was obtained from subjects before data collection.

### Procedures

Potential subjects meeting the research criteria were contacted by a research nurse in the emergency room. Those who agreed to participate were randomly assigned to the comprehensive care, interdisciplinary care, or usual care group. Subjects were randomized by a throw of the dice by a research assistant not involved in the clinical intervention. Subjects were blinded to which intervention they received, but assessors were not blinded.

### Data analysis

Distinct trajectories of PCS and MCS were identified using group-based trajectory models [22–24]. This approach includes two components. First a basic model classifies individuals into groups based on similarities of their trajectories over time. In particular, latent class analysis was used to derive trajectory parameters through maximum likelihood estimation with the following specifications:

1 $$ \mathrm{L}\mathrm{n}{Y}_{iT}^{*g} = {\upbeta_0}^{\mathrm{g}} + {\upbeta_1}^{\mathrm{g}}{\mathrm{Time}}_{\mathrm{iT}} + {\upvarepsilon}_{iT}^{*}\ \mathrm{with}\ \mathrm{i} = 1, \dots \mathrm{n} $$

Ln *Y*_iT_^* *g*^ is a latent variable with a zero-inflated Poisson distribution representing the health status (i.e., either PCS or MCS) of individual i at time T (e.g., 1 month) given membership in group g. Time refers to assessment time from 1 month after discharge. The coefficients β_0_^g^ and β_1_^g^ are associated with the intercept and rate of change in PCS and MCS scores, respectively. ε_iT_^*^ is a disturbance term that is normally distributed with 0 mean and constant variance.

A series of models with two to six groups was examined with the optimal number of groups determined by the Bayesian information criterion (BIC). Within each group, PCS and MCS scores were analyzed as an intercept only, linear or nonlinear model of time, although a linear function is shown in Equation 1 as an illustration. In the second component, trajectory group membership was treated as a dependent variable, which was a function of demographic covariates and treatment interventions, in a fashion similar to that of multinomial logistic regression analysis. In particular, we evaluated the following specifications:

2 $$ {e}^{z_i{\uptheta}_g}\ {\uppi}_{\mathrm{g}}\left({\mathrm{Z}}_{\mathrm{i}}\right) = {e}^{z_i{\uptheta}_g}/{\varSigma}_{\mathrm{g}} $$

where θ_g_ represents the parameters of a multinomial logistic model that captures the effects of predictors z_i_ (e.g., intervention group, attrition, and pre-fracture performance of ADLs) on π_g_, and the probability of membership in group g [24]. Equations 1 and 2 were estimated by an SAS software package, with accompanying Proc Traj [23].

## Results

### Subject characteristics

As indicated in Table 1, the 281 subjects had an average age of 76.36 years (SD = 7.28), with 64.4 % (*n* = 181) being female, 52.7 % (*n* = 148) being married, and 44.1 % (*n* = 124) being illiterate. Before admission, they had on average 2.39 (SD = 1.48) chronic diseases and 67.6 % (*n* = 190) were independent in pre-fracture ADLs. The majority had femoral neck fracture (57.3 %, *n* = 161), 42.7 % (*n* = 120) had trochanteric fracture, 63 % (*n* = 177) received internal fixation of the fracture, and 37 % (*n* = 104) received arthroplasty. Subjects in the experimental and control groups did not differ significantly in any characteristics.

**Table 1:** Demographic characteristics and health-related quality of life of elderly Taiwanese patients with hip fracture (*N* = 281)

Characteristic	Mean (SD)	*n* (%)
Age, years	76.36 (7.28)	
Gender		
Female		181 (64.4)
Marital status		
Married		148 (52.7)
Widowed/divorced		133 (47.3)
Educational background		
Illiterate		124 (44.1)
≥ Primary school		157 (55.9)
Number of comorbidities ^a^	2.39 (1.48)	
Type of fracture		
Femoral neck		161 (57.3)
Trochanteric		120 (42.7)
Type of surgery		
Arthroplasty		104 (37.0)
Internal fixation		177 (63.0)
Pre-fracture independence in ADL		
Yes		190 (67.6)
No		91 (32.4)
PCS score		
Baseline (1 month post discharge)	45.53 (5.92)	
3 months	53.14 (9.40)	
6 months	59.27 (10.42)	
12 months	63.67 (10.88)	
MCS score		
Baseline (1 month post discharge)	55.31 (9.72)	
3 months	55.54 (8.51)	
6 months	53.54 (8.93)	
12 months	51.97 (9.53)	
Attrition		
Baseline (1 month post discharge)		7 (2.49)
3 months		20 (7.11)
6 months		33 (11.74)
12 months		46 (16.37)

*ADL* activities of daily living, *PCS* physical component summary score, *MCS* mental component summary score

^a^ Comorbidities include heart disease, hypertension, stroke, dementia, Parkinson’s disease, diabetes, lung disease, renal disease, liver disease, and cancer

Subjects’ average PCS score was 45.53 (SD = 5.92) at 1 month following discharge and improved to 63.67 (SD = 10.88) at 12 months following discharge. The average MCS score was 55.31 (SD = 9.72) at 1 month following discharge and remained relatively stable during the first 3 months following discharge, but slightly decreased to 51.97 (SD = 9.53) at 12 months following discharge (Table 1).

### Trajectories of PCS and MCS

#### PCS

**Fig. 1:**
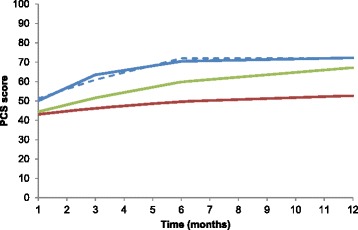
Trajectories of physical component summary scale (PCS) over 12 months after hip fracture-surgery in elderly Taiwanese patients. Solid lines represent observed trajectories; dashed lines indicate predicted trajectories. Red line indicates poor PCS; green line indicates moderate PCS; blue line indicates good PCS

Our analyses identified three PCS trajectories among subjects with hip fracture (Table 2 and Fig. 1)_._ The first trajectory was characterized as poor PCS (*n* = 103, 36.65 %). Subjects in this group experienced a significant, but small improvement in PCS from 43 points to 49 points during the first 6 months after hospitalization, and remained stable at 49 to 52 points from 6 to 12 months following discharge. Similarly, the second trajectory was characterized as moderate PCS (*n* = 96, 34.16 %). Subjects in this group started with a PCS score at 44 points, which improved to 60 during the next 6 months, and remained relatively stable at 67 thereafter (Fig. 1). The third trajectory was described as good PCS (*n* = 82, 29.18 %). Subjects in this group improved substantially from a PCS score of 50 points to 70 points during the first 6 months after discharge and remained relatively stable at 72 points during the following 6 months (Fig. 1).

**Table 2:** Estimated trajectory classes and group-specific growth parameters of health-related quality of life (*N* = 281)

Growth parameter	PCS classes			MCS classes		
	Poor	Moderate	Good	Poor	Moderate	Good
Intercept	3.72^***^	3.71^***^	3.83^***^	3.75^***^	3.95^***^	4.11^***^
Linear slope	0.04^***^	0.08^***^	0.11^***^	-0.010 ^*^	-0.007^**^	-0.005^***^
Quadratic slope	-0.002^**^	-0.004^***^	-0.006^***^			
Group proportion	36.65	34.16	29.18	13.87	29.89	56.23
Alpha0	-21.28			-21.14		
Alpha1	0.26			-0.23		
Alpha2	-0.05					
Model fit statistics						
BIC (*N* =1018)	-3545.68			-3578.91		
BIC (*N* =281)	-3536.67			-3572.48		
AIC fit index	-3511.21			-3554.29		
Log likelihood	-3497.21			-3544.29		

*BIC* Bayesian information criterion, *AIC* Akaike’s information criterion, *PCS* physical component summary score, *MCS* mental component summary score

Level 1, *N* = 1018, and Level 2, *N* = 281

^*^ *p* < 0.05, ^**^ *p* < 0.01, ^***^ *p* < 0.001

#### MCS

**Fig. 2:**
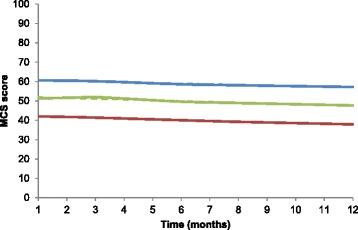
Trajectories of mental component summary scales (MCS) over 12 months after hip-fracture surgery in elderly Taiwanese patients. Solid lines represent observed trajectories; dashed lines indicate predicted trajectories. Red line indicate poor MCS; green line indicates moderate MCS; blue line indicates good MCS

Our analyses identified three linear decreasing MCS trajectories among subjects with hip fracture (Table 2 and Fig. 2). For the first trajectory, poor MCS (*n* = 39, 13.87 %), subjects had a low MCS of 42 points in the 1st month that declined to 38 at the end of 12 months. The second trajectory could be characterized as moderate MCS (*n* = 84, 29.89 %), with the MCS score beginning at 51 in the 1st month and declining to 48 at the end of 12 months following discharge. The third trajectory could be characterized as good MCS (*n* = 158, 56.23 %). Subjects in this group had an average MCS score of 61 at baseline and declined to 57 at the end of 12 months following discharge (Fig. 2).

### Intervention effects on distinct trajectories of PCS and MCS

The interdisciplinary care and comprehensive care models made a significant difference in the PCS trajectories of older Taiwanese subjects with hip fracture. Those who received comprehensive care were 2.69 times more likely to experience good PCS than those who received usual care (*b* = 0.99, odds ratio [OR] = 2.69, CI = 7.24–1.00, *p* = 0.049) (Table 3). Similarly, those who received interdisciplinary care were 3.75 times more likely to experience good PCS than those who received usual care (*b* = 1.32, OR = 3.75, CI = 10.53–1.33, *p* = 0.012) (Table 3). These intervention effects remained robust even after adjusting for pre-fracture ADL performance (*b* = -2.27, OR = 0.10, CI = 0.68–0.02, *p* = 0.018) and attrition (*b* = -0.94, OR = 0.39, CI = 1.49–0.10, *p* = 0.168). In contrast with the effects on PCS trajectories, the interdisciplinary and comprehensive models did not show statistically significant effects on MCS (Table 4).

**Table 3:** Factors associated with health-related quality of life for PCS trajectory group membership

Trajectory	Parameter	β^a^	SE^a^	Odds ratio (95%CI)
Poor PCS	Reference group			
Moderate PCS	Constant	-0.04	0.43	0.96 (2.23–0.42)
	Comprehensive group	0.34	0.47	1.40 (3.54–0.56)
	Interdisciplinary group	0.73	0.49	2.08 (5.46–0.80)
	Attrition	-1.27	1.24	0.28 (3.18–0.02)
	Pre-fracture ADL dependent	-0.49	0.43	0.61 (1.42–0.26)
Good PCS	Constant	-0.63	0.49	0.53 (1.39–0.20)
	Comprehensive group	0.99	0.50	2.69 (7.24–1.00) ^*^
	Interdisciplinary group	1.32	0.53	3.75 (10.53–1.33) ^*^
	Attrition	-0.94	0.68	0.39 (1.49–0.10)
	Pre-fracture ADL dependent	-2.27	0.96	0.10 (0.68–0.02) ^*^
Model fit statistics				
BIC (*N* = 1018)	-3558.53			
BIC (*N* = 281)	-3544.37			
AIC fit index	-3504.35			
Log likelihood	-3482.35			

*BIC* Bayesian information criterion, *AIC* Akaike’s information criterion. Level 1, *N* = 1018, and Level 2, *N* = 281

^a^ Non-standardized

^*^ *p* < 0.05

**Table 4:** Factors associated with health-related quality of life for MCS trajectory group membership

Trajectory	Parameter	β^a^	SE^a^	Odds ratio (95%CI)
Poor MCS	Reference group			
Moderate MCS	Constant	0.94	0.71	2.55 (10.37–0.63)
	Comprehensive group	0.32	0.66	1.38 (5.10–0.38)
	Interdisciplinary group	1.24	0.72	3.46 (14.37–0.83)
	Attrition	-1.83	1.36	0.16 (2.32–0.01)
	Pre-fracture ADL dependent	-1.14	0.67	0.32 (1.19–0.09)
Good MCS	Constant	1.74	0.66	5.70 (20.77–1.57)^**^
	Comprehensive group	0.25	0.48	1.28 (3.28–0.50)
	Interdisciplinary group	0.86	0.61	2.37 (7.88–0.71)
	Attrition	-1.10	0.55	0.33 (0.97–0.11)^*^
	Pre-fracture ADL dependent	-1.17	0.49	0.31 (0.81–0.12)^*^
Model fit statistics				
BIC (*N* = 1018)	-3598.60			
BIC (*N* = 281)	-3587.02			
AIC fit index	-3554.27			
Log likelihood	-3536.27			

*BIC* Bayesian information criterion, *AIC* Akaike’s information criterion. Level 1, *N* = 1018, and Level 2, *N* = 281

^a^ Non-standardized

^*^ *p* < 0.05, ^**^ *p* < 0.01

## Discussion

This study contributes to current knowledge by depicting distinctive prototypical trajectories for both physical and mental HRQoL following a hip fracture and by showing intervention effects for different trajectory groups. These trajectories are more informative than measures at one or two times and average trajectories derived by hierarchical linear modeling because a significant health difference at one time may diminish or even reverse at a later time, and an average trajectory cannot represent differences in changes over time between subjects. Understanding distinct trajectories in physical and mental HRQoL and exploring intervention effects of different care models on specific trajectories may facilitate improvements in managing subjects following hip fracture.

### Distinct trajectories of physical and mental HRQoL following hip fracture

Consistent with our H_1_, both physical and mental HRQoL during the first year after hospitalization for hip fracture followed multiple distinct courses or trajectories that could be characterized as poor, moderate and good trajectories [5]. These trajectories were clinically different, based on the suggested minimal clinically important difference (MCID) for PCS and MCS of 2.5 to 7.8 points [25, 26], and that their confidence intervals were not overlapping and the differences among trajectories were > 10 points. These criteria indicate that the postoperative PCS and MCS trajectories (poor, moderate and good) for older persons with hip fracture differed clinically and represented different patient groups. Distinct trajectories may reflect differences in etiology and thus call for targeted treatments.

### Natural courses of physical and mental HRQoL following hip fracture

This study provides significant findings on the natural history of changes in physical and mental HRQoL after hip fracture by quantifying the levels and rates of change in PCS and MCS over 1 year. For PCS, all three trajectories improved over time, whereas for MCS, all three trajectories declined slightly over time during the first year following discharge. Physical HRQoL of older patients with hip fracture has been shown to improve during the first year following discharge, with improvement most rapid during the first 6 months [27–29]. On the other hand, the slight decline in mental HRQoL is consistent with the high prevalence of depressive symptoms during the first year following hip fracture [30, 31].

### Intervention effects

Our study extends the findings of previous randomized controlled trials on older adults with hip fracture [9, 32] by showing that the intervention effects specifically targeted patients originally in the “poor” PCS trajectory by increasing their likelihood of being in the “good” PCS group. The first trial documented the average beneficial effects of interdisciplinary (referred to as “subacute”) and comprehensive care models on older Taiwanese patients with hip fracture [9]. The second trial showed that older Norwegian patients with hip fracture had significantly better overall improvements in mobility, ADL performance, and QoL for at least 1 year after surgery after receiving comprehensive orthogeriatric care, including comprehensive geriatric assessment and treatment, early discharge planning, early mobilization, and individualized rehabilitation than their counterparts who received usual care on an orthopedic trauma ward [32]. Our analysis showed minimal intervention effects on specific MCS trajectories, similar to the averaged intervention effects [9].

Our study findings indicate that both the interdisciplinary and comprehensive care models can be implemented specifically for subjects with hip fracture and poor physical HRQoL. Subjects with hip fracture and SF-36 PCS scores ≤ 40 might benefit most from interdisciplinary subacute care and comprehensive care including geriatric assessment, 1 year of in-home rehabilitation, supported discharge planning, and management of depressive symptoms, malnutrition management, and fall prevention in improving their physical health-related outcomes.

### Study limitations

The generalizability of the findings are limited to older patients with hip fracture, but without severe cognitive impairment and relatively independent in pre-fracture performance of ADLs due to our sample inclusion criteria. Another limitation is that our study was single blinded; only subjects and families were blinded to the interventions. A third study limitation is that HRQoL was not assessed at baseline, making it difficult to explore the intervention effects more completely. Lastly, the sample size estimated might not support our current hypotheses, because the sample size estimated primarily based on prior intervention effects on physical HRQoL of the interdisciplinary care. The sample estimation did not consider the intervention effects of a comprehensive care mental health related outcomes. Based on the current results, to make the MCS trajectories significant over 1 year following hospital discharge for comprehensive care model, the sample size calculations for latent class analysis is estimated to be 1424 in the future study [33].

## Conclusion

Changes in postoperative HRQoL for people with hip fracture during the first year following hip fracture can be categorized as poor, moderate and good for both physical and mental HRQoL, with physical HRQoL improving and mental HRQoL declining over time. An interdisciplinary care model that included geriatric assessment, supported discharge planning and 4 months of in-home rehabilitation, and a comprehensive care model including management of malnutrition and depressive symptoms as well as fall prevention in addition to interdisciplinary care effectively improved physical health-related outcomes. In particular, these two care models are especially beneficial for people who originally had poor physical HRQoL (for example, PCS ≤ 40) in that these models enhanced their chances of having good physical HRQoL. In other words, these models can be used to target people with hip fracture and initially poor physical HRQoL, thus obtaining optimal effects from the intervention. Finally, even though the interdisciplinary care model provided only 4 months of in-home rehabilitation, compared to the 1 year in-home program provided in the comprehensive care model, it was as beneficial as comprehensive care in improving PCS of people with hip fracture.

### Availability of supporting data

The datasets supporting the conclusions of this article are not available in an open access repository because the authors have not finished the data analysis yet. If anyone is interested in exploring specific issue, please contact Prof. Yea-Ing L Shyu.

## Abbreviations

ADL: Activities of daily living
BIC: Bayesian information criterion
BP: Bodily pain
CBI: Chinese barthel index
GH: General health perceptions
HRQoL: Health-related quality of life
MCID: Minimum clinical importance difference
MCS: Mental component summary scale
MH: General mental health
MNA: Mini nutritional assessment
MOS SF-36: Medical outcomes study short form 36
OR: Odds ratio
PCS: Physical component summary scale
PF: Physical functioning
RE: Role disability due to emotional problems
RP: Role disability due to physical health problems
SF: Social functioning
VT: Vitality
